# Dietary, anthropometric, and biochemical determinants of uric acid in free-living adults

**DOI:** 10.1186/1475-2891-12-11

**Published:** 2013-01-12

**Authors:** Erick Prado de Oliveira, Fernando Moreto, Liciana Vaz de Arruda Silveira, Roberto Carlos Burini

**Affiliations:** 1 Center for exercise metabolism and nutrition (CeMENutri) Department of Public Health Botucatu School of Medicine (UNESP), Botucatu, Brazil; 2 Department of Pathology Botucatu School of Medicine (UNESP), Botucatu, Brazil; 3 Department of Bioestatistic Bioscience Institute (UNESP), Botucatu, Brazil; 4 CeMENutri Departamento de Saúde Pública Faculdade de Medicina UNESP, Distrito de Rubião Jr. s/n°, Botucatu, SP, 18.618-970, Brazil

**Keywords:** Uric acid, Diet, Body composition, Inflammation, Metabolic syndrome components

## Abstract

**Background:**

High plasma uric acid (UA) is a prerequisite for gout and is also associated with the metabolic syndrome and its components and consequently risk factors for cardiovascular diseases. Hence, the management of UA serum concentrations would be essential for the treatment and/or prevention of human diseases and, to that end, it is necessary to know what the main factors that control the uricemia increase. The aim of this study was to evaluate the main factors associated with higher uricemia values analyzing diet, body composition and biochemical markers.

**Methods:**

415 both gender individuals aged 21 to 82 years who participated in a lifestyle modification project were studied. Anthropometric evaluation consisted of weight and height measurements with later BMI estimation. Waist circumference was also measured. The muscle mass (Muscle Mass Index – MMI) and fat percentage were measured by bioimpedance. Dietary intake was estimated by 24-hour recalls with later quantification of the servings on the Brazilian food pyramid and the Healthy Eating Index. Uric acid, glucose, triglycerides (TG), total cholesterol, urea, creatinine, gamma-GT, albumin and calcium and HDL-c were quantified in serum by the dry-chemistry method. LDL-c was estimated by the Friedewald equation and ultrasensitive C-reactive protein (CRP) by the immunochemiluminiscence method. Statistical analysis was performed by the SAS software package, version 9.1. Linear regression (odds ratio) was performed with a 95% confidence interval (CI) in order to observe the odds ratio for presenting UA above the last quartile (♂UA > 6.5 mg/dL and ♀ UA > 5 mg/dL). The level of significance adopted was lower than 5%.

**Results:**

Individuals with BMI ≥ 25 kg/m^2^ OR = 2.28(1.13-4.6) and lower MMI OR = 13.4 (5.21-34.56) showed greater chances of high UA levels even after all adjustments (gender, age, CRP, gamma-gt, LDL, creatinine, urea, albumin, HDL-c, TG, arterial hypertension and glucose). As regards biochemical markers, higher triglycerides OR = 2.76 (1.55-4.90), US-CRP OR = 2.77 (1.07-7.21) and urea OR = 2.53 (1.19-5.41) were associated with greater chances of high UA (adjusted for gender, age, BMI, waist circumference, MMI, glomerular filtration rate, and MS). No association was found between diet and UA.

**Conclusions:**

The main factors associated with UA increase were altered BMI (overweight and obesity), muscle hypotrophy (MMI), higher levels of urea, triglycerides, and CRP. No dietary components were found among uricemia predictors.

## Introduction

Uric acid (UA) is a waste product of the human purine balance. It is formed by adenosine, inosine, hypoxanthine, adenine and guanine [1]. Additionally, UA is the main hydrophilic antioxidant in our organism [2] as it is responsible for up to 2/3 of the total antioxidant capacity in human blood [3]. UA exerts a protective action on vitamins C and E [4] and inhibits free radicals, such as radical peroxyl and peroxynitrite, thus protecting cell membrane and DNA [5,6]. However, while acute increases seem to provide antioxidant protection, chronic UA increases are associated with higher risk for coronary artery disease (infarction) [7-9].

High urate concentrations are associated with articular deposits and the onset of (gout) arthritis [10], metabolic syndrome (MS) [11], and with cardiovascular diseases [12]. It is known that anthropometric parameters, dyslipidemia, hypertension, inflammation, and insulin resistance can increase the UA concentration [12].

Other confounding factor is the diet and the relationship between diet and UA has not been fully elucidated, since most studies do not measure urate basal concentrations, do not exclude confounding factors and do not correctly evaluate ingested nutrients [13]. However, some studies shows that the diet can influence the high UA concentration, whereas a positive association is observed among high intake of meat and seafood and a negative association with the dairy product intake is observed [13-15].

Some confounding factors can influence the hyperuricemia because several variables are associated with each other [16-19], what makes it difficult to know which component can influence isolated the UA concentration, for example, adiposity markers are associated with higher insulin resistance and leptin production, and both reduce renal uric acid excretion, thus increasing its concentration. HDL-c concentration is negatively associated to insulin resistance, and is found an indirect association with uric acid concentration. Furthermore, obese individuals usually presents metabolic syndrome diagnostic, which can also increase uric acid serum concentrations due to synthesis increase (triglycerides concentration) and lower excretion (hypertension). Many dietary factors are associated with metabolic syndrome components and also can influence indirectly the urate. Additionally, obesity and muscle mass (MM) reduction are associated with low-intensity chronic inflammation, and uric acid levels can increase in order to protect the organism against the moderate oxidative stress resulting from this situation [20].

Uric acid may be beneficial (antioxidant) or deleterious in high concentrations. Hence, the manegement of UA serum concentrations is promising in the treatment of certain diseases [21]. Therefore, diet, body composition and factors related to the metabolic syndrome are determinant in UA increase, but no studies have gathered all these factors or conjointly evaluated these components in order to learn which of them are actually mainly responsible for UA increase.

Hence, the present study aimed to evaluate what are the main factors associated with higher uricemia values, analyzing diet, body composition and biochemical markers.

### Individuals

A descriptive cross-sectional study was conducted in a subgroup of participants clinically screened for the lifestyle modification program “*Mexa-se Pró-Saúde* [Move for Health]” from 2002 to 2006. This program is offered to patients with non-communicable chronic diseases and consists of regular physical exercise and nutritional counseling. The *Center for Physical and Nutritional Metabolism* (CeMENutri) has conducted this program in Botucatu since 1992. Botucatu is a city located in mid-São Paulo State, approximately 230 km west of the capital city. It has a population of 121,274 habitants [22].

The inclusion criteria for participants were individuals of both genders with at without metabolic or motor disabilities that would limit physical exercise.

A convenience sample was consisted of 1,075 individuals who were 51.6 ± 10.2 years old, and 59% of them were females. All the subjects signed a free-consent form, and the research project was approved by the Research Ethics Committee (document no. CEP 3272–2009) of the Botucatu School of Medicine (FMB), Univ Estadual Paulista - UNESP, Brazil. Of the 1,075 subjects, 415 had biochemical, anthropometric and dietetic data.

### Dietary intake

Dietary intake data was determined by using 24-hour recalls. The diet was documented by trained professionals, and in order to obtain accurate information, the subjects were asked how often they usually ate during the day, what food varieties were consumed, how food was prepared, what the serving size was, and what food/meal brands were consumed. The diets were analyzed by NutWin® software (2002), version 1.5 [23], and the main nutrients of interest were energy, protein, fat (saturated, mono and polyunsaturated), cholesterol, carbohydrates, and dietary fiber. Mean individual nutrient intakes per day were computed using the NutWin database and Brazilian food tables [24-26]. The Healthy Eating Index (HEI) modified for the Brazilian population was used to assess the quality of the participant’s diet [27]. The original HEI was developed based on a 10-component system of five food groups with a total possible index score of 100. This method was adapted for the Brazilian population based on the Brazilian food guide, which has eight food groups and 12 components to measure food intake variety. Each of the 12 components has a score ranging from 0 to 10; therefore the total possible index score is 120.

### Anthropometry

Body weight was measured by a platform-type anthropometric scale (Filizola®) with a maximum capacity of 150 kg and an accuracy of 0.1 kg. Height was determined by a portable Seca® stadiometer with accuracy of 0.1 cm [28]. By using body-weight and height measurements, the Body Mass Index (BMI) was calculated.

Waist circumference (WC) was measured at the point midway between the last rib and the iliac crest. A steel Sanny® anthropometric tape measure (without a lock) was used for all measurements.

A bioelectrical impedance device (Biodynamics®, model 450, USA) was used to determine body fat percentage (% body fat) [29] and body-muscle mass, whose data were used for calculating the muscle-mass index (MMI) [30].

### Clinical evaluation of arterial blood pressure

Systolic and diastolic arterial blood pressure was evaluated with the individual in the seated position according to the procedures described by the VI Brazilian Guidelines on Arterial Hypertension [31]. Values of systolic blood pressure ≥ 130 mm Hg and/or diastolic blood pressure ≥85 mm Hg were considered to be abnormal [32,33].

### Biochemical analyses

Blood samples were collected after overnight fasting for 10 to 12 hours using a vacuum venous puncture. The individuals were previously instructed to not perform vigorous physical exercise 24 hours and/or not drink alcohol 72 hours before collection. Laboratory analyses of lipid parameters (total cholesterol, fractions and triglycerides), glucose, uric acid, urea, gamma-glutamyl transferase (γ-GT), albumin and total proteins were performed within 4 hours after blood collection using the Dry- Chemistry method (Vitros® system, Johnson & Johnson) while C-reactive protein (CRP) was measured by a high-sensitive Chemiluminescent method (Siemens Diagnostics), at the Clinical Analyses Laboratory of the Botucatu School of Medicine University Hospital - UNESP in Botucatu/SP. Low density lipoprotein (LDL-c) was calculated by Friedewald equation (LDL-c = TC- (HDL-c + TG/5) [34]. Glomerular filtration rate was calculated [35] and added in adjustments.

### Metabolic syndrome

Diagnosis of the metabolic syndrome was performed according to the NCEP-ATP III criteria [32,33] with adaptation for glucose values [36]. The 5 components used were high systolic and diastolic blood pressure and WC measurements, and plasma levels of triglycerides, HDL-c and, glucose. The metabolic syndrome was diagnosed when 3 or more of these components were abnormal.

### Operational definition of variables

Higher uric acid was considered when was higher than the 4^th^ quartile (♂ > 6,5 mg/dL and ♀ > 5 mg/dL). Overweight was classified as BMI ≥ 25 kg/m^2^[37], altered WC was considered when above 102 cm (40.16 inches) for men and above 88 cm (34.65 inches) for women [32,33]. Low muscle mass was defined by the muscle mass index (MMI) (lower quartile vs. higher quartile). Higher body fat was defined by body fatness higher than 25% for men and higher than 35% for women [38]. Hypertriglyceridemia was defined by plasma concentrations ≥ 150 mg/dL [32,33], lower HDL-c as < 40 mg/dL for men and < 50 mg/dL for women [32,33] and hypercholesterolemia was > 200 mg/dL [39]. Higher plasma glucose was defined by ≥ 100 mg/dL [36], higher CRP and gamma-gt (4^th^ quartile).

### Statistical analysis

Statistical analyses were conducted by using the SAS software for windows (SAS version 9.1.3., SAS Institute, Inc., Cary, NC) and STATISTICA 6.0. Sample normality was tested by the Shapiro-Wilk test. Descriptive statistics were performed for the study, and continuous variables are presented as means ± standard deviation (SD) or median and interquartile intervals. UA values were divided into quartiles for both genders, and one-way ANOVA and generalized linear model considering gamma distribution and link function log were performed in order to observe the difference between variables according to the increase in the UA quartile. Logistic regression (Odds ratio) was made in order to determine the probability of UA increase (♂UA > 6.5 mg/dL and ♀ UA > 5 mg/dL) by food intake, anthropometry and biochemical analysis. Backward stepwise multiple regression analysis was used to determine the main components responsible for UA increase according to gender. A *p* value < 0.05 was adopted as significant.

## Results

Higher UA concentrations (last quartile) were associated with higher values for body adiposity markers (weight, BMI, WC and % body fat) whilst lower UA concentrations (Q1) were associated with higher MMI values when compared to the other quartiles. There was no difference in relation to the individuals’ age or height (Table 1).

**Table 1:** Demographic, anthropometric, clinical and laboratory characterization of individuals according to the uric acid quartile

	**Quartile 1**	**Quartile 2**	**Quartile 3**	**Quartile 4**
**Age (years)**	53,4 ± 9.7^a^	52.7 ± 9.8^a^		51.7 ± 9.5^a^	54.1 ± 9.4^a^
**Weight (kg)**	69.3 ± 13.3^a^	71.5 ± 12.7^a^		76.8 ± 16^b^	81.6 ± 17^c^
**Height (m)**	1.62 ± 0.08^a^	1.61 ± 0.08^a^		1.62 ± 0.1^a^	1.63 ± 0.08^a^
**Body Mass Índex (kg/m**^**2**^**)**	26.17 ± 3.7^a^	27.2 ± 3.9^a^		29.0 ± 5.2^b^	30.5 ± 5.2^c^
**Waist Circumference (cm)**	87.64 ± 11.9^a^	90.9 ± 11.1^b^		94.2 ± 13^c^	99.4 ± 13.7^d^
**% of body fat**	27.8 ± 5.6^a^	29.3 ± 7.8^ab^		31 ± 8.4^b^	33.6 ± 8.5^c^
**Muscle Mass Índex (kg/m**^**2**^**)**	10.8 ± 4.1^a^	8.8 ± 4^b^		8.7 ± 3.9^b^	8.8 ± 4^b^
**Total Energy Intake (kcal)**	1612 ± 608^a^	1584 ± 603^a^		1651 ± 700^a^	1581 ± 613^a^
**Carbohydrate (%)**	51.05 ± 9.7^a^	51.7 ± 10.8^a^		50.8 ± 9.1^a^	51 ± 10.7^a^
**Protein (%)**	18.8 ± 6.11^a^	19.1 ± 6.2^a^		19.3 ± 6.2^a^	18.4 ± 5.9^a^
**Total Lipid (%)**	30.17 ± 8.9^a^	29.2 ± 9.2^a^		30.0 ± 8.1^a^	30.4 ± 8.5^a^
**Saturated Lipid (%)**	8.12 ± 4.1^a^	7.7 ± 3.6^a^		8.4 ± 4.1^a^	7.9 ± 3.6^a^
**Monounsaturated Lipid (%)**	8.32 ± 2.95^a^	9.0 ± 4.3^a^		8.9 ± 3.5^a^	9.1 ± 3.5^a^
**Polyunsaturated Lipid (%)**	5.65 ± 2.8^a^	6.2 ± 2.8^a^		6.1 ± 3.3^a^	7.4 ± 3.6^b^
**Fiber (g)**	14.1 ± 7.1^a^	13.8 ± 8.6^a^		13.3 ± 7.6^a^	13.1 ± 8.1^a^
**Cholesterol (mg)**	183 ± 120.8^a^	188 ± 126^a^		198 ± 124^a^	185 ± 135^a^
**Cereal (servings)**	3.35 ± 1.8^a^	3.5 ± 1.6^a^		3.4 ± 1.8^a^	3.4 ± 1.5^a^
**Fruits (servings)**	3 ± 2.73^a^	2.8 ± 3.1^a^		2.7 ± 3.2^a^	2.8 ± 3.0^a^
**Vegetables (servings)**	3.6 ± 2.8^a^	2.9 ± 2.5^ab^		2.2 ± 2^b^	3.1 ± 3.1^a^
**Legumes (servings)**	1.2 ± 1.7^a^	1.1 ± 1.1^a^		1.1 ± 1.5^a^	1.3 ± 1.8^a^
**Dairy (servings)**	1.7 ± 1.7^a^	1.4 ± 1.2^a^		1.5 ± 1.2^a^	1.5 ± 1.3^a^
**Meat (servings)**	2.13 ± 1.6^a^	2.3 ± 1.6^a^		2.1 ± 1.5^a^	1.9 ± 1.5^a^
**Sugar (servings)**	1.67 ± 1.6^a^	1.7 ± 1.7^a^		2.0 ± 1.9^a^	1.5 ± 2.2^a^
**Oil (servings)**	2.4 ± 2.4^a^	2.4 ± 2.5^a^		2.4 ± 1.9^a^	2.7 ± 2.2^a^
**Variety (number)**	14.27 ± 4.2^a^	13.3 ± 3.5^a^		13.1 ± 3.6^a^	13.5 ± 4^a^
**Health Eat Index (points)**	86.4 ± 13^a^	83.2 ± 12.5^a^		82.3 ± 14.3^a^	81.8 ± 15^a^
**Plasma urea (mg/dL)**	30.5 ± 8.5^a^	32 ± 10.6^ab^		32.2 ± 10.2^ab^	34.8 ± 14.4^b^
**Plasma creatinine (mg/dL)**	0.89 ± 0.2^a^	0.93 ± 0.2^ab^		0.96 ± 0.19^b^	1.04 ± 0.3^c^
**Plasma HDL-c (mg/dL)**	60.2 ± 18^a^	52.3 ± 15.5^b^		49.3 ± 14.3^b^	49.4 ± 13.9^b^
**Plasma LDL-c (mg/dL)**	123 ± 35.2^a^	132 ± 36.1^ab^		134 ± 37.4^b^	134 ± 38^b^
**Plasma glucose (mg/dL)**	101 ± 28^a^	102 ± 37^a^		100 ± 32^a^	104 ± 30^a^
**Plasma total Cholesterol (mg/dL)**	208 ± 41^a^	213 ± 44^ab^		215 ± 40^ab^	220 ± 41^b^
**Plasma triglycerides (mg/dL)**	114 (81–171)^a^	117 (88–174)^ab^		138 (93–183)^bc^	177 (124–232)^c^
**Plasma total Protein (g/dL)**	7.08 ± 0.5^a^	7.36 ± 0.6^b^		7.5 ± 0.6^b^	7.52 ± 0.6^b^
**Plasma albumin (g/dL)**	4.51 ± 0.48^a^	4.4 ± 0.5^a^		4.4 ± 0.5^a^	4.4 + 0.5^a^
**Plasma gamma-gt (l/U)**	25 (18–32.5)^a^	27 (19–57)^ab^		29 (21–44)^ab^	34 (25.5-51.5)^b^
**Plasma calcium (mg/dL)**	8.8 ± 0.4^a^	8.9 ± 0.9^a^		9.1 ± 0.4^a^	9.1 ± 0.4^a^
**Plasma c-Reactive Protein (mg/dL)**	0.14 (0–0.3)^a^	0.2 (0.1-0.75)^a^		0.26 (0.1-0.5)^a^	0.3 (0.18-0.6)^a^
**Systolic blood pressure (mmHg)**	126 ± 17^a^	126 ± 18^ab^	132 ± 17^b^	130 ± 17^ab^	
**Diastolic blood pressure (mmHg)**	80 ± 11^a^	79 ± 11^a^	82 ± 10^a^	83 ± 11^a^	

Different letters = P < 0.05; a,b,c= Anova one-way.

As regards dietary intake, greater polyunsaturated fat intake was observed in individuals with higher UA quartiles. Smaller intake of vegetables was also observed in the second and third quartiles. The intake of other macronutrients and groups of the pyramid servings did not show difference according to the increase in the UA quartile (Table 1).

Urea, creatinine, LDL-c, TC, TG, total proteins and gamma-GT showed lower values according to the increase of the UA quartiles. The opposite was observed for HDL-c, with individuals in the first quartile showing higher concentrations. No significant difference was observed for the other biochemical parameters. Sistolyc blood pressure showed a higher value in the third quartile while there was no difference for diastolic blood pressure (Table 1).

The odds ratio for the UA at the last quartile (♂ UA > 6.5 mg/dL and ♀ UA > 5 mg/dL) was performed, according to alterations in body composition. In model 1, additionally to the adjustment for gender and age, all body composition components were included in the same model, because it was possible to observe the influence of each compartment alone. Individuals with BMI, WC and % body fat above normality as well as with little muscle mass (MMI < *P*25) showed higher chances of high uric acid. When MS was adjusted for BMI, % body fat and MMI (Model 2) remained significant, and WC analysis was not performed, since it is part of the MS criteria. The four body-composition components evaluated remained significant when MS was removed from the adjustment and CRP (inflammation) gamma-gt LDL-c, glomerular filtration (creatinine and urea) were added. When the MS components (HDL-c, TG, hypertension and glucose) were added to the adjustment, only high BMI and low muscle mass remained significant (Table 2).

**Table 2:** Odds ratio to present the UA at the higher quartile (UA ♂ > 6.5 mg/dL and ♀ UA > 5 mg/dL) according to body composition

	**Model 1**	**Model 2**	**Model 3**	**Model 4**
**Body Mass Index (abnormal vs normal)**	2.23 (1.32-3.75)*	2.81 (1.5-5.28)*		1.97 (1.41-3.41)*	2.28(1.13-4.6)*
**Muscle Mass Index (P25 vs P75)**	8.11 (4.52-14.58)*	17.51 (7.98-38.48)*		7.16 (3.6-14.29)*	13.4 (5.21-34.56)*
**Waist Circumference (abnormal vs normal)**	2.21 (1.39-3.52)*	-		1.91 (1.15-3.16)*	1.14 (0.6-2.17)
**% body fat (abnormal vs normal)**	1.96 (1.22-3.16)*	1.8 (1.03-3.16)*		1.76 (1.04-2.98)*	1.54 (0.81-2.9)

Model 1 – Adjusted for gender, age and other components of body composition analyzed.

Model 2 – Model 1 + Metabolic Syndrome.

Model 3 – Model 1 + c-Reactive Protein, gamma-gt, LDL-c, creatinine, urea and albumin.

Model 4 – Model 3 + HDL-c, triglycerides, hypertension and glucose.

*p < 0.05.

As regards dietary intake, intake >60% of carbohydrate of total caloric intake when compared to <50% of CHO, represented higher chances of UA increase (Model 1). It is known that high CHO intake can increase glycemia and/or TG, and these may be related to UA increase. Hence, both were adjusted (Models 2 and 3). When high CHO intake was adjusted for glycemia or TG, the effect of CHO on UA was lost. Thus CHO intake could directly influence UA serum concentrations, firstly increasing glycemia or TG (Table 3). The intake of food pyramid servings and HEI did not show influence on UA (data not shown).

**Table 3:** Odds ratio to present the UA at the higher quartile (UA ♂ > 6.5 mg/dL and ♀ UA > 5 mg/dL) according to dietary intake

	**Model 1**	**Model 2**	**Model 3**
**Carbohydrate ( >60% vs < 50%)**	3.22 (1.01-10.27)*	1.38 (0.22-8.73)	1.43 (0.23-9.0)
**Protein (>15% vs <15%)**	0.99 (0.45-2.18)	1.05 (0.36-3.05)	0.83 (0.28-2.45)
**Total Lipid (>35% vs 25-35%)**	1.57 (0.62-4.02)	1.02 (0.27-3.86)	0.88 (0.23-3.36)
**Saturated lipid (>10% vs <10%)**	0.77 (0.35-1.70)	0.32 (0.10-1.0)	0.34 (0.11-1.06)
**Monounsaturated Lipid (>10% vs <10%)**	1.38 (0.64-2.98)	2.51 (0.71-8.89)	2.02 (0.56-7.34)
**Polyunsaturated Lipid (>10% vs <10%)**	1.87 (0.82-4.29)	2.05 (0.62-6.82)	2.51 (0.75-8.39)
**Cholesterol (>300 vs <300 mg)**	1.51 (0.61-3.76)	1.99 (0.33-11.95)	1.25 (0.20-7.83)
**Fiber (Q4 vs Q1)**	1.06 (0.44-2.55)	1.99 (0.45-8.77)	1.51 (0.33-6.85)

Model 1 – adjusted for gender, age, Body Mass Index, Total Energy Intake and other components of diet (macronutrient) analyzed.

Model 2 – Model 1 + glucose.

Model 3 – Model 1 + triglycerides.

*p < 0,05.

The biochemical data were gathered in the same model, and adjusted for gender and age (Model 1) was added. The altered values for creatinine, HDL-c, LDL-c and TG, conjointly with values in the last quartile for gamma-gt and CRP were significant in increasing the chances of individuals’ showing high UA. When body composition was added to the adjustment (Model 2), the creatinine and HDL-c values lost their effect; however, gamma-gt, LDL-c and TG remained significant. MS and glomerular filtration rate were also added to the adjustment (Model 3); TG remained significant, and the last CRP quartile and altered urea values became significant, thus increasing the chances of UA increase. Hypertension and glomerular filtration rate were added to the model, and MS was removed (Model 4) because, in this way, all MS components would be gathered in the same adjustment model, and no modifications were observed in relation to the latter model, that is, altered urea and TG values; and values above the CRP last quartile were associated with UA increase (Table 4).

**Table 4:** Odds ratio to present the UA at the higher quartile (UA ♂ > 6.5 mg/dL and ♀ UA > 5 mg/dL) according to biochemical data

	**Model 1**	**Model 2**	**Model 3**	**Model 4**
**Creatinine (abnormal vs normal )**	3.25 (1.09-9.69)*	1.99 (0.47-8.37)	0.83 (0.12-5.69)	0.83 (0.12-5.67)
**Urea (abnormal vs normal)**	1.25 (0.84-1.86)	1.35 (0.75-2.41)	2.72 (1.27-5.83)*	2.53 (1.19-5.41)*
**Albumin (Q4 vs Q1)**	1.4 (0.9-2.21)	0.74 (0.38-1.42)	1.05 (0.41-2.72)	1.11 (0.43-2.87)
**HDL-c (abnormal vs normal)**	1.57 (1.12-2.17)*	1.21 (0.77-1.90)	0.98 (0.53-1.81)	1.17 (0.66-2.08)
**Gamma-gt (Q4 vs Q1)**	4.58 (2.22-9.44)*	3.41 (1.08-10.7)*	3.53 (0.91-13.61)	3.33 (0.87-12.73)
**Glucose (abnormal vs normal)**	1.05 (0.76-1.44)	1.01 (0.64-1.57)	1.03 (0.56-1.87)	1.27 (0.74-2.18)
**LDL-c (abnormal vs normal)**	1.4 (1.04-1.88)*	1.52 (1.02-2.28)*	1.16 (0.71-1.90)	1.15 (0.70-1.89)
**C-Reactive Protein (Q4 vs Q1)**	2.37 (1.43-3.91)*	1.81 (0.91-3.58)	2.87 (1.10-7.45)*	2.77 (1.07-7.21)*
**Triglycerides (abnormal vs normal)**	1.83 (1.34-2.50)*	2.44 (1.59-3.83)*	2.29 (1.21-4.30)*	2.76 (1.55-4.90)*

Model 1 – adjusted for gender, age and other biochemical components analyzed.

Model 2 – Model 1 + Body Mass Index, Muscle Mass Index, waist circumference and% body fat.

Model 3 – Model 2 + Metabolic Syndrome and glomerular filtration rate.

Model 4 – Model 2 + Hypertension and glomerular filtration rate.

*p < 0,05.

The main factors of each compartment were gathered in order to observe which factor showed greater influence on UA serum concentrations according to gender. It was observed that WC and creatinine increase, together with HDL-c and muscle mass reduction, is 36% responsible for UA increase in females. In males, however, BMI increase and muscle mass decrease showed 28% responsibility (Table 5).

**Table 5:** Analysis of backward stepwise multiple regression of variables according to UA concentrations

**Female**							**Male**						
	Betaβ	EP	B	EP	t	p		Betaβ	EP	B	EP	t	p
**intercept**			−0.638	0.713	−0.894	0.372	**intercept**			1.709	0.783	2.183	0.031
**Waist Circumference (cm)**	0.475	0.057	0.051	0.006	8.303	0.000	**Body Mass Index (kg/m**^**2**^**)**	0.526	0.081	0.189	0.029	6.538	0.000
**Muscle Mass (kg)**	−0.393	0.057	−0.040	0.006	−6.925	0.000	**Muscle Mass (kg)**	−0.415	0.081	−0.041	0.008	−5.152	0.000
**Creatinine (mg/dL)**	0.242	0.047	2.244	0.440	5.100	0.000							
**HDL-c (mg/dL)**	−0.197	0.049	−0.016	0.004	−4.046	0.000							
**R**^**2**^	0.36					0.00	**R**^**2**^	0.28					0.00

## Discussion

The main finding of the study was that higher UA levels were associated positively with BMI, triglycerides, urea, and CRP and inversely with MMI. According to the gender, the main predictors for UA increase were BMI and muscle mass for men. Waist circumference, creatinine, and muscle mass (positively); and HDL-c (negatively) were associated for women.

UA serum concentrations are maintained through the synthesis and excretion of urate [40]; hence, creatinine and serum urea, which are considered to be glomerular function markers, are directly related to UA concentrations, particularly due to the urate excretion relationship. Choe *et al.*, 2008 [41] reported that creatinine has strong influence on urate concentrations. Rathmann *et al.* (2007) investigated UA concentrations in a population consisting of black and white adults for ten years and identified an independent relationship of creatinine and urate [42].

Creatinine serum concentration was one of the main UA-related factors for females. In the odds-ratio model (Table 5), altered creatinine increased the chances of hyperuricemia, but this fact occurred only in the first adjustment of the model. When the body-composition components were added, the effect was lost. On the other hand individuals with altered urea showed approximately 2.5 more chances of increasing UA, regardless of the influence from other factors, including glomerular filtration rate, which means that mechanisms independently of excretion can influence this relation.

The increase in uricemia distribution quartiles did not follow age increase. It is usually reported that, as age advances, a gradual reduction in glomerular filtration occurs, which results in plasma retention of solutes that are normally excreted by the kidneys [43]. In the present study, age ranged from 21 to 82 years, but most individuals were 40 to 60 years old and probably showed little variability in glomerular filtration. Additionally, the adjustment for glomerular filtration rate was made.

The increase in uricemia distribution quartiles was accompanied by increase in body adiposity markers (weight, BMI, WC and % body fat). Altered WC was positively associated with urate concentrations; however, when fitted for other MS components, the effect was lost, thus showing that WC is indirectly associated with UA, since individuals with abdominal adiposity could present MS and/or alteration in its components, and these could be responsible for affecting UA. It is believed that TG is the main MS component influencing UA, as it was the only factor that remained significant after all adjustments.

Other studies showed the relationship between WC or abdominal adiposity and UA increase [44,45], reporting that visceral fat is more related to UA increase than subcutaneous fat [46,47]. Adipose tissue produces various cytokines, including leptin, and the probable explanation for the association between WC and hyperuricemia would be the association found by Bedir *et al*., (2003) [48] and Fruehwald-Schultes *et al*., (1999) [49], where UA serum concentrations are independently related to leptin. Two mechanisms could explain such relationship. The first would be the influence of leptin on the renal function, which decreases renal UA excretion. In the second mechanism, UA could modulate leptin concentrations, thus increasing its gene expression or decreasing its excretion [49].

BMI above 25 kg/m^2^ was one of the main components associated with UA increase both in the fitted models and in the multiple regression analysis for males, and it also showed significant correlation with uricemia. BMI showed a positive relation with leptin concentrations [50], which is a factor leading to UA increase.

Additionally, individuals with high BMI may show insulin resistance, TG alteration and high blood pressure, and all these factors are related to UA increase [12]. In our study, insulin sensitivity was not measured; however, the analyses were fitted for SAH and TG, and the values remained significant. This showed that independent mechanisms from these two factors could explain such relation, probably to insulin resistance.

UA increase is observed in individuals with insulin resistance, probably because hyperinsulinemia would cause lower renal UA excretion [51]. Besides, insulin could also indirectly affect UA, since there is an association between hyperinsulinemia and hypertriglyceridemia.

In the present study, individuals with altered TG showed approximately 2.5 more chances of UA increase, regardless of other variables (body composition. gender, inflammation, dyslipidemia, MS, and SAH). Some studies show that high plasma triglyceride concentrations are related to hyperuricemia [52-55]. One of the explanations for such relation would be that, during the TG synthesis, there would be a greater need for NADPH for the *de novo* synthesis of fatty acids [53]. Matsuura *et al.* (1998) report that the synthesis of fatty acids in the liver is related to the *de novo* synthesis of purine, thus accelerating UA production [45].

In the present study, UA concentrations were negatively determined by MM (kg) in males and females. The hypothesis would be the negative correlation existing between MM and inflammation [56], since an association between UA increase and inflammatory markers has been reported [57]. However, in our study, muscle mass was fitted for inflammation (CRP), and the influence of muscle mass on UA remained. Possibly, other mechanisms would influence the association between muscle reduction and UA increase, such as oxidative stress.

A recent study observed an inverse relation between MMI and UA in healthy individuals older than 40 years [58]. The authors believe that increased urate serum concentrations would be a causal factor for sarcopenia, especially through increased inflammation and oxidative stress [58]. During the sarcopenic process, reactive oxygen species (ROS) and oxidative stress increase, and one of the mechanisms for ROS increase would be the activation of the xanthine oxidase metabolic pathway, which increases UA production and the superoxide radical [59]. In the present study, oxidative stress was not evaluated, and it may be a causal factor for such relation between UA and MMI; however, further studies analyzing the cause and effect between these two factors and their main mechanisms are necessary.

A positive association was found between UA and CRP (inflammation), regardless of body composition (hyperadiposity and/or sarcopenia), gender, age and the presence of MS and its components, which means that there are a direct relation between these two factors. Another study showed a positive association between inflammation and UA, but no adjustment was performed to observe whether the effect would remain the same [60]. *In-vitro* studies showed that UA exerts a pro-inflammatory effect, thus stimulating the production of interleukin-1, interleukin-6 the tumor necrosis factor [61].

Individuals with higher CRP concentrations showed increased UA; however, UA is positively associated with total plasma antioxidant capacity, thus showing beneficial effects. On the other hand, deleterious relations of urate, such as the inverse association with adiponectin and the positive relation with E-selectin, were previously observed [62]. Such associations can show that UA may increase in order to enhance total plasma antioxidant capacity against moderate oxidative and inflammatory stress, thus being a protective feature against factors related to cardiovascular diseases. It is also noteworthy that UA may be deleterious in high concentrations [21], which shows the importance of maintaining urate values within normality.

Reduced HDL-c was one of the main factors responsible for UA increase (negative association) in females. This fact was observed by the backward stepwise multiple regression analysis. The negative correlation between HDL-c and AU was previously described [60], and it has been recently shown that the higher the urate concentration, the smaller the size of HDL-c and LDL-c particles, which provides a greater atherogenic profile [60]. However, our group has shown that, when the adjustment for body composition and SM components is performed, the association between UA and HDL-c is lost [16], which was also observed in the present study after adjustment. The probable mechanisms for the inverse association between these two factors would be the relation existing between HDL-c reduction and insulin resistance [63].

It is expected that individuals with high UA will have more chances of presenting MS [52]. MS is associated with increased oxidative stress [64], and it known that UA is a potent antioxidant [3]. Thus, it can be supposed that urate increase could result from the defense mechanism from such oxidative stress [65]. These factors further enhance our results, since the factors associated with UA (BMI, MMI, urea, TG and CRP) remained significant even after adjustment for MS.

In the present study, diet did not show direct influence on UA, but inadequate diet, conjointly with lack of physical activity, could alter body composition (higher adiposity), and such alteration would change UA. Additionally, an indirect relation of high carbohydrate intake was observed through the possible alterations in TG and/or glycemia.

The intake of protein, meat and legumes, which could be related to increased purine intake, was not related to UA concentrations, thus agreeing with the literature [13,14]. Some studies showed that high purine intake does not influence UA, since a purine-rich diet would be responsible for increasing only 1 to 2 mg/dL of UA [66,67]. Although the present study evaluated the intake of protein-source foods, an exclusive investigation on purine-source foods was not performed. Hence, further studies are required in order to learn about the actual effects of the intake of purine-rich foods on urate concentrations.

A significant weak and inverse correlation was observed (r = −0.11) between the intake of dairy products and UA, but when fitted for gender, BMI and VCT, the significance was lost. Although the present study did not observe such relation, other investigations reported an inverse association between dairy products and UA [13,14,68], which is explained by the fact that milk proteins (lactalbumin and casein) showed a uricosuric effect [69].

Based on our data, lifestyle-modification conducts targeted at lean-mass maintenance and fat-mass reduction and concern about dietary composition are suggested in primary care for uricemia increase in non-uremic individuals.

### Study limitations

This was a cross-sectional study, and some cause/effect relationships cannot be possibly confirmed, but only whether or not an association between the studied factors exists. The dietary intake investigation on the individuals was performed only on one day, and a food recall for at least three weekdays would ideal, since there may be dietary variations that were not analyzed. Some dietary factors were not evaluated, such as the intake of alcohol, purine and caffeinated drinks, which are known for their interference with UA values [14,68,70]. Insulin resistance was not measured, and that would be important for this type of analysis, since some UA increase mechanisms are involved with this factor. Furthermore, medication intake and smoking status were not controlled.

## Conclusions

The main factors associated with UA increase were altered BMI (overweight and obesity), muscle hypotrophy (MMI), higher levels of urea, triglycerides, and CRP. No dietary components were found among uricemia predictors.

## Abbreviations

BMI: Body mass index; MMI: Muscle Mass Index; WC: Waist circumference; NCEP-ATPIII: National Cholesterol Education Program-Adult Treatment Painel III; MS: Metabolic Syndrome; γ–GT: γ-glutamyl transferase; CRP: C-reactive protein; TG: Triglycerides.

## Competing interests

The authors declare that they have no competing interests.

## Authors’ contributions

EPO wrote the manuscript, FM corrected the manuscript, LVAS made the statistical analysis and corrected the manuscript. RCB read, corrected, and approved the final version of the manuscript. All authors read and approved.
